# Preparation and Characterization of Antimony-Doped Tin Oxide Nanocrystallite Coatings on 316L Stainless Steel

**DOI:** 10.1155/2021/9402537

**Published:** 2021-11-03

**Authors:** Giang Nguyen Truong, Long Hoang Vinh, Suong Huynh Thu, Phuong Nguyen Thi Hong, Bac Nguyen Quang

**Affiliations:** ^1^Department of Chemistry, Faculty of Materials, National University of Civil Engineering, No. 55 Giai Phong Rd., Hanoi, Vietnam; ^2^School of Chemical Engineering, Hanoi University of Science and Technology, No. 1 Dai Co Viet St., Hanoi, Vietnam

## Abstract

The study on the coatings containing antimony-dope tin oxide mostly has been done on titanium and its alloys for preparation of electrode materials. In this study, we try to prepare the similar coatings on 316L stainless steel, which is more common than titanium, and investigate some characteristics of the formed coatings. The preparation of the coating is by the impregnation of the substrate in solution containing SnCl_4_ and SbCl_3_ with the ratio of 93/7, and the concentration of SbCl_3_, 10 g/L in isopropanol, pH of 1–1.2, and the annealed temperature of 450°C give suitable coating for electrode materials. Some coating features, such as the crystal structure (XRD), morphology (SEM), hardness, electrical resistant, and electrochemical behaviors of the coating on the substrate have been also investigated. The results of the study show that the coatings can be used as electrode materials for the effective treatment of organic colorants in water such as methylene blue in water treatment.

## 1. Introduction

The mixed metal oxides on metal substrate attracted many researchers due to their scientific meanings and possible applications [1]. The SnO_2_ coatings doped with Sb have been studied and applied as an anode electrode in color wastewater treatment especially on titanium metal substrate [2–6]. In the system of Sb-doped SnO_2_ coatings on titanium, the crack-mud phenomenon, i.e., the crack of the coatings during drying and heat treatment, often occurred that makes the life of the electrode potentially reduced due to the electrolyte permeation through cracks or coating damage [3].

Stainless steel alloys are more common than titanium, with high mechanical strength, high chemical resistance, and good electrical conductivity, and they can be used as the substrate for coating and preparation of electrode materials. However, to the best of our knowledge, the investigation and application of antimony-doped tin oxide coatings on high steel alloys are extremely rare. In this study, the preparation and characterization of Sb-doped SnO_2_ coatings on 316L stainless steel will be investigated in detail. The coatings were prepared by impregnating the substrates into a solution containing SnCl_4_ and SbCl_3_ and then annealed at designated temperature. The morphology and crystallinity of the coatings were determined by scanning electron microscopy (SEM) and X-ray diffraction (XRD). The electrochemical properties of this electrode were studied by cyclic voltammetry. The coating has been applied as the anode for the electrochemical degradation of methylene blue in solution.

## 2. Experimental

### 2.1. Reagents and Materials

Tin tetrachloride pentahydrate (SnCl_4_.H_2_O 98.0%), antimony chloride (SbCl_3_), isopropanol, (C_3_H_8_O, 99.5%), and hydrochloric acid (HCl, 37%) are all from Sigma Aldrich for experiments. Acetone (C_3_H_6_O, 99.7%, Xylong, China) is used for cleaning electrode. All chemicals are used as received without any further purification.

Samples of commercial 316L stainless steel with the reported composition of C (≤0.03%), Mn (≤2%), P (≤0.045%), S (≤0.03%), Si (≤1%), Cr (16–18%), Ni (10–14%), Mo (2–3%), and iron in balance are supplied by Chau Duong International Industrial Co., Ltd.

### 2.2. The Preparation of the Electrode

The 316L stainless steel plates with a size of 12 × 12 × 2 mm were polished with abrasive papers, cleansed with water and acetone, and then dried. The coating solution was prepared by dissolving 132.86 g SnCl_4_.5H_2_O and SbCl_3_ (0–15 g) in isopropanol, pH of the solution was adjusted to 0.8–1.2 by concentrated hydrochloric acid, and then diluted into 1 L of solution with isopropanol. The prepared solution is heated to about 90°C, and then, the samples are dipped for 2 minutes, dried, and calcined at designated temperatures from 250 to 600°C in a muffle oven for an hour. This procedure was repeated 6 times to obtain a suitable coating thickness. Finally, the electrode was annealed for 5 hours in the final step to induce crystallization of oxide mixture.

### 2.3. Characterization and Analysis Methods

The crystal structure and compositions of the coating film were studied by an X-ray diffractometer (XPERT PRO, Netherlands) using CuK*α* radiation in the 2*θ* range of 20^o^–70^o^. The surface morphology of the metal oxide coating and the qualitative analysis of elements present in the coatings were measured on scanning electron microscopy (SEM, JEOL, JSM-7600F, US).

The resistivity of the film was measured by the four-probe method. Electrochemical properties of the formed film were investigated by open circuit potential measurement vs. time and potentiodynamic polarization curves in the 2 g/L Na_2_SO_4_ solution at room temperature. The electrochemical measurements were conducted on a three-electrode electrochemical system (Autolab PGSTAT 302N, Netherlands) with the prepared electrode served as the working electrode, Pt mesh as the counter electrode and a saturated calomel electrode as the reference electrode. The working electrode potentials were scanned from −0.5 V to +1.5 V versus open circuit potential with a scanning rate of 5 mV/s for potentiodynamic polarization measurement.

The UV-Vis absorption behavior of the MB solution is measured on a Cary 100 equipment, recorded from 300 nm to 800 nm at room temperature. Discoloration efficiency in methylene blue (MB) solution treatment is evaluated as the relative peak area calculated from 450 to 750 nm from the UV-Vis spectra.

## 3. Results and Discussion

### 3.1. Effect of Different Annealing Temperatures on Coating Structure and Surface Morphology

**Figure 1:**
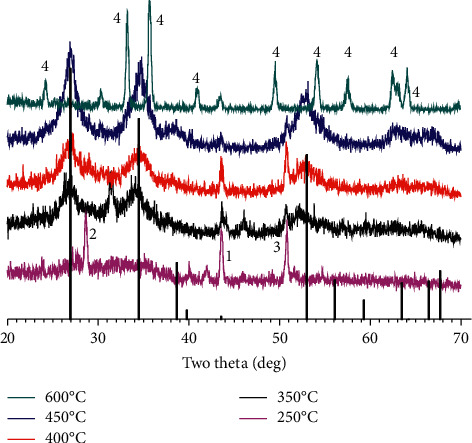
XRD patterns of SnO_2_-doped Sb coatings on 316L stainless steel at various annealing temperatures. The peaks of SnO_2_ (adapted from [7]) are presented as the vertical black bars and **1,** NiO (JCPDS 78-0643); **2,** Sb_2_O_3_ (JCPDS 42-1466); **3,** Sb_2_O_4_ (JCPDS 37-0854); **4,** Cr_2_O_3_ (JCPDS 84-1616).

The formation of the coating is completed when the deposited is annealing. The heat treatment is chosen to decompose the reactants on the substrate. The formation of the coating will depend on the decomposition of the precursors and the interaction of the particles. The results of the crystal structure investigation of the coating with the various annealing temperature are shown in Figure 1.

Figure 1 shows that the phase of the coating on the substrate is almost the same as the one of SnO_2_ when the samples are annealed at various temperatures from 350 to 450°C. At high annealing temperature, such as 600°C, chromium oxide phase (JCPDS 84-1616) and mixture of nickel and iron oxides are formed as indicated by the presence of different peaks from two theta of 20–70°. The peaks for iron oxides are very weak or unclear, probably because of its fluorescence, so that the suitable temperature for heat treatment will be around 450°C.

The XRD patterns also show that the crystallinity of the coating may not be very high as indicated by low intensity of most XRD peaks and the noise of the background. The small crystal may favor the formation of the smooth surface when coated with the amorphous portion.

It is noted that the presence of the Sb species on the coating is not indicated by the XRD data due to the incorporation of the Sb species on the SnO_2_ structure host. Only a small peak at around 51° is assigned to the one of Sb_2_O_3_. The higher the annealing temperature, the lower the peak of Sb_2_O_3_, probably due to the formation of antimonite or antimonate rather than the oxide of antimony. Because the ionic radii of Sb and Sn are rather close, the partial replacement of Sn by Sb may occur to some extent as expected, and some properties of the coating will be improved such as the coating conductivity, via supra.

**Figure 2:**
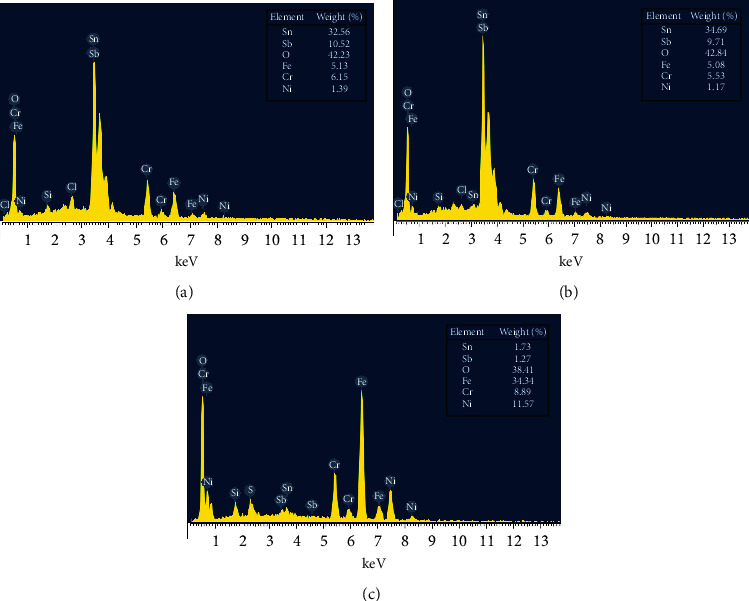
EDX patterns of Sb-doped SnO_2_ coatings on 316L stainless steel at various annealing temperatures: (a) 350°C; (b) 450°C, and (c) 600°C.

The qualitative analysis and the evaluation of coating composition are further investigated by the EDX measurement. The results of the analysis on the surface of the samples annealed at 350, 450, and 600°C are shown in Figure 2.

Figure 2 and the corresponding analysis inset confirm the presence of Sn and Sb in the samples. The presence of the iron, nickel, and chromium species may come from the substrate materials. When the samples are impregnated into high acidity solutions, some of the materials may be dissoluted and deposited back to the coating. The data analysis shows that the ratio of Sn and Sb is not perfectly the same as the one in the doping solution probably due to the peak positions of Sn and Sb elements are rather close in the EDX spectra, so the evaluation of elemental composition will only give rough numbers.

When the sample is annealed at 600°C, the composition of the coating is totally different from that of the lower one. The analysis data showed that the Sn and Sb contents in the coating are only few percent, and the coating composes mostly of iron, chromium, and nickel oxides. These results are consistent with the results of phase analysis. It means that the beneath layer on the substrate has been corroded or destroyed when dipping or heating the samples.

**Figure 3:**
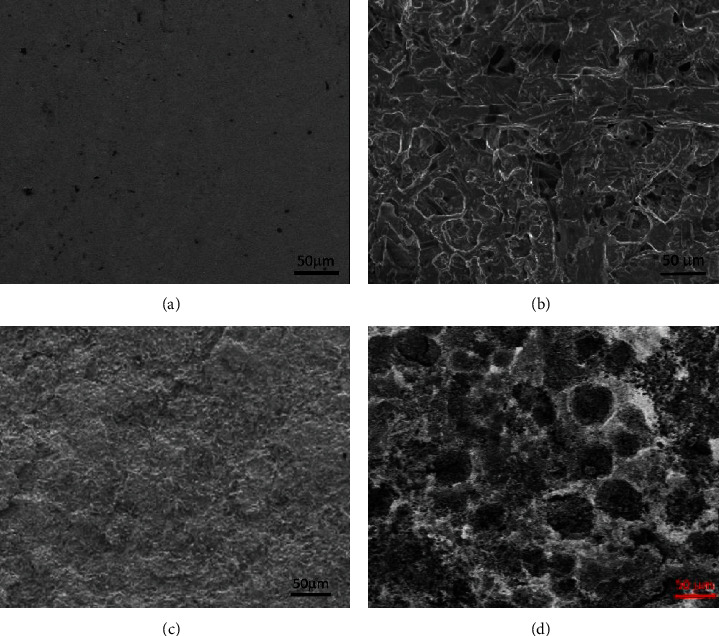
The SEM images of the SnO_2_-doped Sb thin films on (a) 316L stainless steel, (b) 350°C annealing (c), 450°C annealing (d), and 600°C annealing.

The morphology of the samples treated at various temperatures is also studied. The photos of the SEM measurement of the bare substrate and the coatings annealed at 350, 450, and 600°C are shown in Figure 3.

The SEM images in Figure 3 show that the original substrate has a smooth surface, the deposition of the materials on the surface is clear, and the coating morphology is very different from that of the substrate.

At 350°C, the coatings seem to coarse with the formation of large particles with clear edges. The array of the particles makes the coating difficult to be dense to completely cover the substrate surface. Mechanical properties of the coating are rather poor. When the samples anneal at 450°C, the surface of the coating is very smooth. No large particles or grain boundaries are observed, and its physicochemical properties are also improved which maybe suitable for the applications of electrode materials.

The case of the samples annealed at 600°C, and the surface of the samples becomes inhomogeneity with the formation of large lumps of material next to some white ones. The coating seems to be burned out and easily removed from the surface with mechanical force. From the basic structure, composition, and morphology of the coating, it is clear that the suitable annealing temperature is around 450°C and should not be heated to 600°C or higher.

### 3.2. Effect of pH Solution on Coating Structure and Surface Morphology

**Figure 4:**
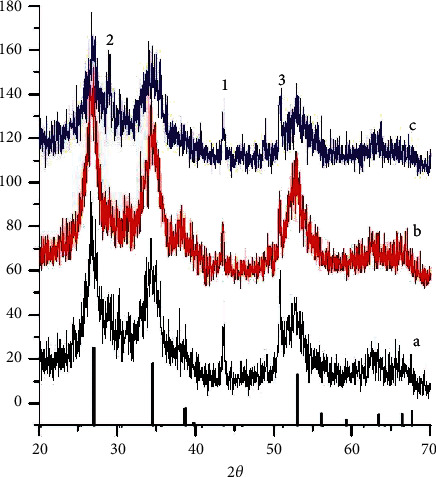
XRD patterns of SnO_2_-doped Sb thin films on 316L stainless steel at different pH solutions. (a) pH = 1.2, (b) 1.0, and (c) 0.8. The peaks of SnO_2_ (adapted from [7]) are present as the vertical black bars on the bottom.

The composition of the dipping solution will influence to the formation and properties of the coating on the substrate surface. The investigation of the pH of the solution on the crystal structure of the coating has been done, and the results are shown in Figure 4.

The results of XRD measurements on the prepared coatings show that at pH 0.8, the coatings are very poor and due to the corrosion of the substrate layer by acid, which may favor the exfoliation of the coatings. The products of corrosion such as iron or other ingredient compounds may be incorporated into the coatings.

At higher pH values of the dipping solution such as pH = 1 or 1.2, the coating is formed with high quality and no exfoliation occurred, and the XRD data show the peaks for Sb-doped SnO_2_ and those of Sb_2_O_3_. When the pH of the dipping solution is increased, the white precipitate of unknown materials formed on the surface of the coating, which may be the metal hydroxides of hydrated oxides. Therefore, the suitable pH value of the solution for dipping will be around 1–1.2.

### 3.3. Electrical Volume Resistivity of the Coating

**Figure 5:**
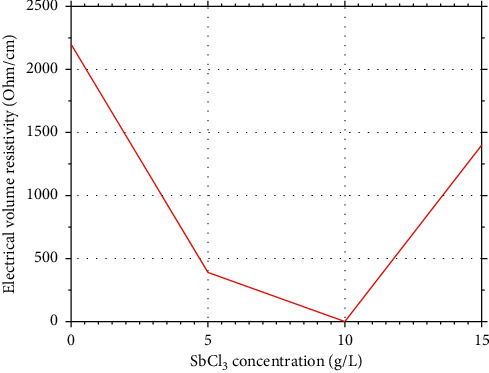
Electrical volume resistivity vs. Sb concentration in the reactant mixture of particles after annealing at 450°C for 5 h.

The incorporation of the Sb oxide on to the Sn oxide has been confirmed by the investigation of the resistivity of the coating at various contents of Sb in the dipping solution. The results of the study and the influence of the Sb content in the solution on the resistivity of the coating are shown in Figure 5.

Figure 5 shows that the coating of SnO_2_ can be formed on the surface of the substrate; however, the resistivity is very high (about 2200 Ω/cm) due to the electricity isolation behavior of SnO_2_.

When adding Sb into the dipping solution, the coating of SnO_2_ containing Sb will be formed with the charge difference of Sn and Sb, and the resistivity of the coating will become much lower. Depending on the content of Sb replaced by Sn in the coating, the resistivity may be improved in a wide range. For example, when the Sb content in the solution is 5%, the resistivity of 390 Ω/cm is observed, the Sb content is increased to 10%, and the value of 0.043 Ω/cm is measured. However, when the Sb content is further increased, the resistivity tends to be increased to the value of 1400 Ω/cm for the solution containing 15% SbCl_3_. This is the fact that, the replacement of Sn by Sb only occurs to some levels, too high content of Sb in the coating may favor the formation of antimonite or antimonate rather than incorporated into the SnO_2_ structure. The content of SbCl_3_ in the dipping solution is around 10 g/L which will give the good conductivity of the coatings.

The coating prepared in this study has very good mechanical properties. The investigation of the hardness shows that the bare substrate only has the value of 217 kg/mm^2^, whereas one of the coatings obtains 475 kg/mm^2^.

### 3.4. Application of Prepared Electrode for Treatment of Methylene Blue

**Figure 6:**
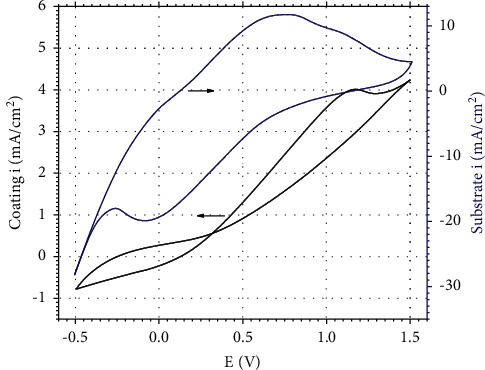
The cyclic voltammetry of the bare 316L stainless steel (blue curve) and the 316L/SnO_2_-Sb_2_O_3_ (black curve) electrodes in solution containing MB 15 mg/L (with 2 g/L Na_2_SO_4_).

The prepared coating on the 316L stainless steel has been applied as the electrode materials for the treatment of methylene blue (MB), a typical organic colorant by the electrochemical oxidation method. The cyclic voltammetry (CV) measurement results of the solution containing 15 mg/L of MB and 2 g/L Na_2_SO_4_ for the 316L stainless steel with and without the coating are shown in Figure 6.

The CV measurement results show that the CV behavior of the bare 316L stainless steel in MB solution is very close to the one in acidic solution [8]. The high value of current density at 0.75 V is ascribed to breakdown of the passive film and the initiation of corrosion of the steel electrode. When the electrode is coated with mixed oxide, the CV measurement shows an oxidation peak at the potential of 1.18 V with the oxidation current of 4.01 mA/cm^2^ during the treatment of MB. The low current density is obtained due to the ionic conductivity behavior of the mixed oxide. However, the oxidation peak is rather broad, probably due to the oxidation of MB may be undergone several steps with the formation of some intermediates, and then, the intermediates are also oxidized to final products as shown in other works [9].

**Figure 7:**
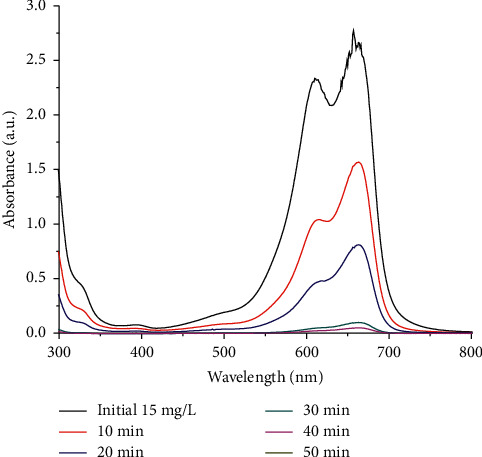
UV-Vis spectra of MB solution at different electrolysis time.

For evaluation of the MB treatment, the UV-Vis spectra of the initial and treated solution vs. duration of electrolysis are shown in Figure 7, and MB discoloration efficiency is given in Table 1.

The results of UV-Vis absorption measurement show that the spectra are corresponding to the one of MB which agrees well with the results in other works and the intermediates, if any, have not detected in the measured range [10]. The content of MB decreased sharply when electrolysis occurred as indicated by the discoloration efficiency which is calculated from the relative peak area ratio. After about 30 min, more than 90% of the original MB has been treated, and the color of the treated solution becomes pale blue. After 50 min of electrolysis, 99.97% of MB has been decomposed, and the solution becomes almost colorless. This experiment indicated that the coating on 316L stainless steel can be used as the electrode for the treatment of a variety of colorants or dyes in water treatment.

## 4. Conclusions

The coatings containing antimony-doped tin oxide on 316L stainless steel have been prepared and structurally characterized XRD, SEM, and CV data measurement. The impregnation of the substrate into the solution contains Sb^3+^ and Sn^4+^ ions with the concentration of SbCl_3_ about 10 g/L, and the pH in the range of 1–1.2; then, annealing at temperature of 450°C can avoid crack-mud phenomena in the preparation of the electrode materials. The prepared electrodes can be used as anode for the electrochemical system in the treatment of organic colorants such as methylene blue or other similar ones. The study on the treatment of MB in solution shows that most of MB has been destroyed within one hour. The fast and complete oxidation of the organic colorants will give a great chance for their application as advanced materials in water treatment.

## Figures and Tables

**Table 1:** Effect of electrolysis time on MB discoloration efficiency.

Electrolysis duration (min)	10	20	30	40	50
Discoloration efficiency (%)	51.28	76.84	97.84	99.08	99.97

## Data Availability

No data were used to support this study.
